# Assessing the solubility of three resin-based luting cements in artificial saliva

**DOI:** 10.12669/pjms.42.1.13111

**Published:** 2026-01

**Authors:** Muhammad Adeel Ahmed

**Affiliations:** 1 Muhammad Adeel Ahmed Department of Restorative Dental Sciences, College of Dentistry, King Faisal University, Al-Ahsa, Saudi Arabia

**Keywords:** Artificial saliva, pH, Panavia SA, Resin cements, Solubility, Resin-modified glass ionomer cement

## Abstract

**Objectives:**

The present study aimed to assess and compare the solubility of three resin-based luting cements in artificial saliva under varying pH conditions.

**Methodology:**

This in-vitro experimental study was conducted at the College of Dentistry, King Faisal University, Al Ahsa, Saudi Arabia, over a period of two months. A total of 45 disc-shaped specimens (8mm diameter, 4mm thickness) were prepared using cylindrical molds: 15 each of Self-Adhesive Methacrylate-Based Resin Cement (Non-MDP), Self-Adhesive MDP-Based Resin Cement, and Resin-Modified Glass Ionomer Cement. The specimens were light-cured for 40 seconds using an LED curing unit (450 mW/cm²) and finished with Sof-Lex discs. Each group was further subdivided, with five specimens immersed in artificial saliva at pH 3.4, pH 6.7, and distilled water at pH 7.0, respectively, for four weeks. Solubility was determined by calculating the mass loss of each specimen after immersion in the test solutions for four weeks. One-way ANOVA and Tukey’s HSD post-hoc test were applied for group comparisons at a significance level of p < 0.05.

**Results:**

The solubility of all three cements was significantly higher in acidic artificial saliva (pH 3.4), with Self-Adhesive MDP-Based Resin Cement demonstrating the lowest solubility (0.022 ± 0.010 mg/mm³), and both Self-Adhesive Methacrylate-Based Resin Cement (Non-MDP) and RMGIC showing similarly high solubility values (0.08 ± 0.021 mg/mm³ and 0.08 ± 0.005 mg/mm³, respectively). No significant difference was observed among the groups at pH 6.7 and pH 7.0.

**Conclusion:**

The solubility of resin-based luting cements is influenced by pH, with significantly greater degradation under acidic conditions. Self-Adhesive MDP-Based Resin Cement exhibited superior resistance, making it more suitable for low pH environments.

## INTRODUCTION

The longevity and retention of dental restorations are significantly influenced by the physical characteristics of luting agents, particularly their solubility. Among these characteristics, solubility plays a pivotal role in determining the clinical effectiveness and durability of luting cements used in restorative procedures.^1^ These materials function by occupying the space between the restoration and the tooth surface, not only withstanding occlusal forces but also minimizing microleakage. However, luting cements positioned at the restoration margins are continuously exposed to the oral environment, making them susceptible to dissolution over time.^1^

Solubility and disintegration of dental cements in saliva or other oral fluids significantly affect the durability and clinical performance of luting agents. Hydrolytic breakdown can lead to the loss of key components, debonding of restorations, microleakage, and recurrent decay. Factors influencing solubility include cement composition, powder-liquid ratio, fluoride release, patient oral hygiene, dentifrice composition, and the pH of saliva, food, and beverages.^2^

Several studies have indicated that a decrease in oral pH often associated with the frequent consumption of acidic beverages significantly increases the solubility and disintegration of dental cements.^3^ This is primarily due to the reduction in salivary pH and the release of protic acids from such drinks, which together contribute to the hydrolytic degradation of the cement matrix. Prolonged exposure to low pH further exacerbates this breakdown process, leading to compromised restoration integrity.^4^

Luting cements are also challenged by various intraoral factors such as masticatory and parafunctional stresses, salivary contamination, fluctuating pH levels, and temperature changes, all of which can degrade their performance.^5^ According to the American Dental Association (ADA), two key properties for evaluating luting materials are water sorption and solubility.^6^ Scientific evidence supports that when resin-based cements are exposed to water or saliva, hydrolytic degradation of weak polymer bonds (such as Van der Waals forces) occurs. This degradation compromises the structural and mechanical integrity of the material by plasticizing the resin polymer, ultimately weakening the bond strength and reducing functional longevity.^7^ Although water absorption may mitigate polymerization shrinkage stress, it does so at the expense of mechanical robustness.^8^

Currently, no single luting cement exhibits all the ideal properties required for universal clinical application. Optimal characteristics include low film thickness, extended working time, controlled setting reaction, low solubility, and minimal microleakage. Zinc phosphate (ZP), resin-modified glass ionomer cements (RMGIC), and resin composite cements are noted for having low film thickness.^7^,^9^ Meanwhile, resin-based and GIC cements offer comparatively longer working times than ZP and RMGI.^10^,^11^ Resin-based cements also exhibit superior fracture toughness, enhanced flexural modulus, and minimal interaction with moisture. Interestingly, GICs have a thermal expansion coefficient and modulus of elasticity that closely resembles that of natural tooth structure. Moreover, resin cement and GIC generally show lower solubility and microleakage than ZP and RMGIC.^10^-^13^ Their solubility tends to increase in the presence of reduced pH levels, raising concerns over their long-term stability.

The oral environment is dynamic and varies in pH, temperature, and salivary composition, all of which can influence the solubility and durability of luting cement. Resin-based luting cements, commonly used for fixed restorations, must withstand these conditions to ensure long-term clinical success. However, limited data exists on their solubility under simulated oral conditions. Consequently, this study was intended to assess and compare the solubility of three resin-based luting cements: Self-Adhesive Methacrylate-Based Resin Cement (Non-MDP), Self-Adhesive MDP-Based Resin Cement, and Resin-Modified Glass Ionomer Cement in artificial saliva environments at varying pH levels. It was hypothesized that all cements would show higher solubility in acidic environments, with MDP-based resin cement being the most resistant.

## METHODOLOGY

This in-vitro comparative experimental study was carried out at the College of Dentistry, King Faisal University, Al Ahsa, Saudi Arabia, for a duration of two months. A total of 45 disc-shaped specimens (8mm in diameter and 4mm in thickness) were fabricated using preformed cylindrical molds. Fifteen molds were filled with Self-Adhesive Methacrylate-Based Resin Cement [Non-MDP] (SeT, SDI limited, Victoria, Australia), another 15 with Self-Adhesive MDP-Based Resin Cement (Panavia SA, Kuraray Noritake Dental Inc, Tokyo, Japan) and the remaining 15 with Resin Modified Glass Ionomer Cement (RIVA, SDI limited, Victoria, Australia). Each mold was sealed with a transparent strip to avoid air entrapment, and a 1-2mm thick glass slide was placed on top to ensure a flat surface. The specimens were then light-cured for 40 s using an LED curing unit (450 mW/cm²) through the glass slide and strip, followed by polishing with Sof-Lex discs.

After curing, the discs were categorized into three groups for further analysis. The SeT Group (Self-Adhesive Methacrylate-Based Resin Cement [Non-MDP]) consisted of 15 discs, with five discs each immersed in artificial saliva at pH 3.4, artificial saliva at pH 6.7, and distilled water at pH 7. Similarly, the Panavia SA Group (Self-Adhesive MDP-Based Resin Cement) included 15 discs, also distributed across the same three storage media and pH conditions. The RMGIC Group (Resin Modified Glass Ionomer Cement) followed the same pattern, with five discs immersed in each of the three solutions-artificial saliva at pH 3.4, artificial saliva at pH 6.7, and distilled water at pH 7, completing the total of 45 discs analyzed in the study. The chemical composition of the artificial saliva included purified water, glycerin, xylitol, PEG-60, hydrogenated castor oil, VP/VA copolymer, flavor, sodium benzoate, xanthan gum, methylparaben, sodium saccharin and cetylpyridium chloride.

**Fig.1:**
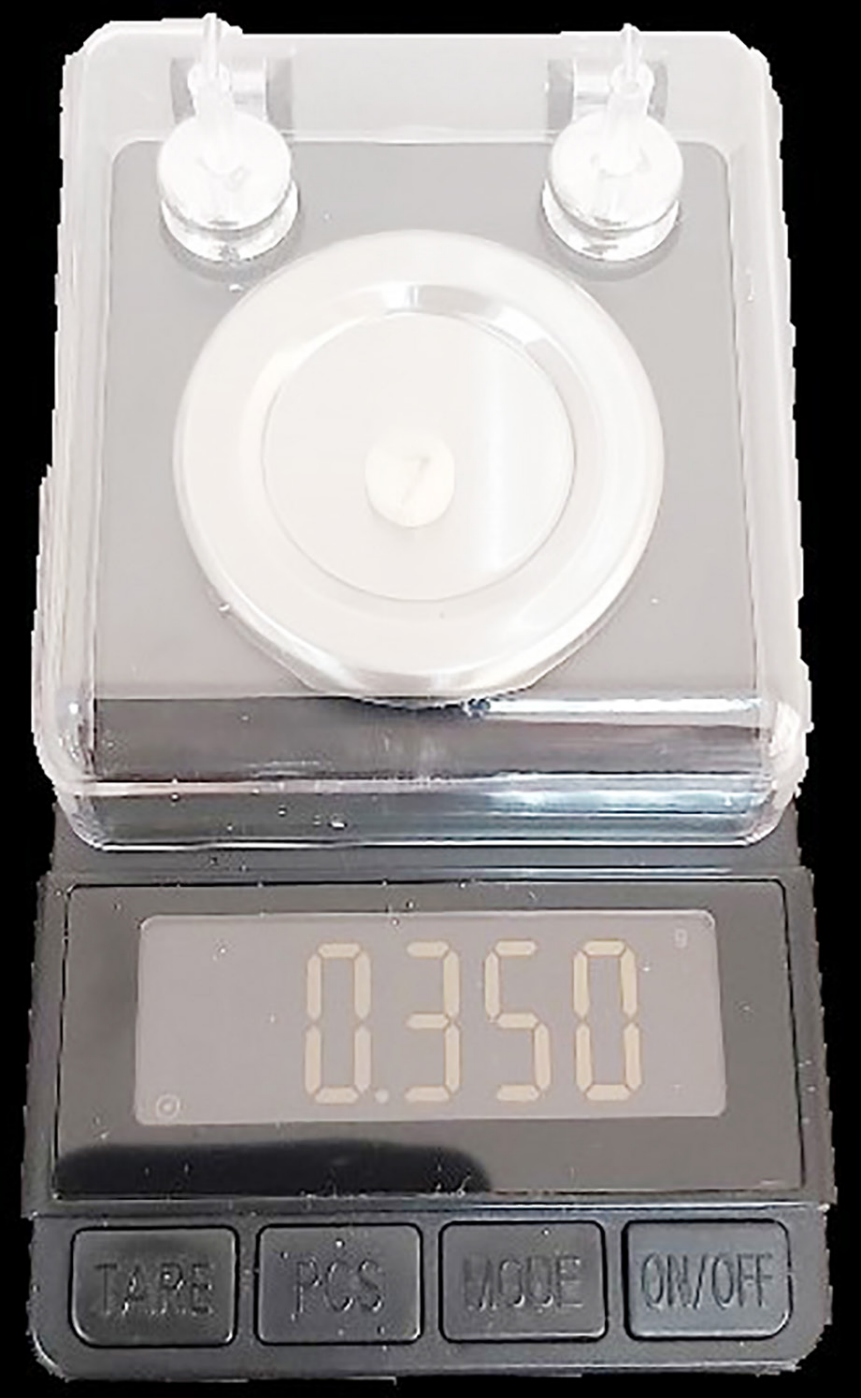
A calibrated digital milligram scale to measure weight.


The initial weight of each specimen was measured using a calibrated digital milligram scale (Maxus, China) Fig.1. The calibration of the digital milligram scale was performed according to the manufacturer instructions using 20gm of calibrated weight in each pan. The specimens were then immersed in separate containers containing artificial saliva at different pH levels and distilled water (humidity of 100% at 37°C) for four weeks. Following immersion, the samples were removed, gently dried, desiccated, and reweighed. Solubility was determined using the ISO standard formula.

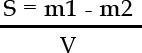


S = Solubility in µg/mm³

m1 = Initial mass of dry specimen

before immersion (µg or mg)

m2 = Final mass of specimen after

immersion and drying (µg or mg)

V = Volume of the specimen in mm³

### Statistical analysis:

Statistical analysis was performed using SPSS version 22.0. Data were assessed for normality using the Shapiro-Wilk test. As the data were normally distributed, one-way analysis of variance (ANOVA) was applied to compare the solubility values of the three luting cements across different pH levels. Post-hoc pairwise comparisons were conducted using Tukey’s Honestly Significant Difference (HSD) test to identify specific group differences. The significance level of p < 0.05 was considered statistically significant.

## RESULTS

The solubility of the three luting cements was assessed under different pH conditions-artificial saliva at pH 3.4 and 6.7 and distilled water at pH 7.0. At pH 3.4, a statistically significant difference in solubility was observed among the groups (*p* < 0.001). Both the SeT group (0.08 ± 0.021 mg/mm³) and the RMGIC group (0.08 ± 0.005 mg/mm³) demonstrated substantially higher solubility compared to the Panavia SA Cement group (0.022 ± 0.010 mg/mm³). However, at pH 6.7 and pH 7.0, no statistically significant differences were observed between the groups (*p* = 0.861 and *p* = 0.372, respectively), as depicted in Table-I.

**Table-I:** Comparison of solubility of three luting cements in artificial saliva and distilled water at different pH levels.

Different pH	Solubility (mg/mm^3^)			
	SeT Group (n=15) Mean ± SD	Panavia SA Group (n=15) Mean ± SD	RMGIC Group (n=15) Mean ± SD	p-value
Artificial saliva (pH 3.4)	0.080 ± 0.021	0.022± 0.010	0.080 ± 0.005	<0.001
Artificial saliva (pH 6.7)	0.004 ± 0.004	0.006 ± 0.002	0.005 ± 0.006	0.861
Distilled water (pH 7.0)	0.030 ± 0.006	0.045 ± 0.010	0.040 ± 0.005	0.372

Pairwise comparisons were evaluated of the solubility differences among the three luting cements under varying pH conditions. In highly acidic artificial saliva (pH 3.4), significant differences were observed between the SeT and Panavia SA cement (p < 0.001), as well as between Panavia SA cement and RMGIC groups (p < 0.001). However, no significant difference was found between the SeT and RMGIC groups (p = 0.974), as both exhibited similarly high solubility. Under neutral conditions (artificial saliva at pH 6.7), no statistically significant differences were observed in any of the comparisons: SeT vs. Panavia SA Cement (p = 0.851), SeT vs. RMGIC (p = 0.938), and Panavia SA Cement vs. RMGIC (p = 0.977). Similarly, in distilled water (pH 7.0), all comparisons showed no significant differences. The solubility values for SeT vs. Panavia SA Cement (p = 0.438), SeT vs. RMGIC (p = 1.000), and Panavia SA Cement vs. RMGIC (p = 0.438) were statistically comparable, as depicted in Table-II.

**Table-II:** Pairwise comparison (Tukey HSD) of solubility among luting cements under varying pH conditions.

Comparison of luting cements at different pH condition		Solubility (mg/mm^3^)	
		Mean ± SD	p-value
Artificial saliva (pH 3.4)	SeT Group vs Panavia SA Group	0.080 ± 0.021 vs 0.022± 0.010	<0.001
	SeT Group vs RMGIC Group	0.080 ± 0.021 vs 0.08 ± 0.005	0.974
	Panavia SA Group vs RMGIC Group	0.022± 0.010 vs 0.080 ± 0.005	<0.001
Artificial saliva (pH 6.7)	SeT Group vs Panavia SA Group	0.004 ± 0.004 vs 0.006 ± 0.002	0.851
	SeT Group vs RMGIC Group	0.004 ± 0.004 vs 0.005 ± 0.006	0.938
	Panavia SA Group vs RMGIC Group	0.006 ± 0.002 vs 0.005 ± 0.006	0.977
Distilled water (pH 7.0)	SeT Group vs Panavia SA Group	0.030 ± 0.006 vs 0.045 ± 0.010	0.438
	SeT Group vs RMGIC Group	0.030 ± 0.006 vs 0.040 ± 0.005	1.000
	Panavia SA Group vs RMGIC Group	0.045 ± 0.010 vs 0.040 ± 0.005	0.438

Intragroup comparisons were performed to evaluate luting cement’s solubility variations across different pH environments. For the SeT, statistically significant differences in solubility were observed between all pH levels. Solubility at pH 3.4 was significantly higher compared to both pH 6.7 and pH 7.0 (p < 0.001 for both comparisons), while a significant difference also existed between pH 6.7 and pH 7.0 (p = 0.030), indicating that this material is highly sensitive to pH changes, especially in acidic conditions. In the Panavia SA Cement group, solubility at pH 3.4 was significantly greater than at pH 7.0 (p = 0.004) and pH 6.7 (p = 0.040). Additionally, a highly significant difference was found between solubility at pH 6.7 and pH 7.0 (p < 0.001). For the RMGIC group, solubility at pH 3.4 was significantly higher compared to both pH 6.7 and pH 7.0 (p < 0.001 for both). Likewise, a significant difference was also noted between pH 6.7 and pH 7.0 (p < 0.001), as depicted in Table-III.

**Table-III:** Intragroup solubility comparisons for each luting cement at different pH levels.

Type of luting cement	Solubility	Artificial saliva (pH 3.4)	Artificial saliva (pH 6.7)	Distilled water (pH 7)
SeT Cement	artificial saliva (pH 3.4)	―	<0.001	<0.001
	artificial saliva (pH 6.7)	<0.001	―	0.030
	distilled water (pH 7)	<0.001	0.030	―
Panavia SA Cement	artificial saliva (pH 3.4)	―	0.040	0.004
	artificial saliva (pH 6.7)	0.040	―	<0.001
	distilled water (pH 7)	0.040	0.040	―
Resin modified GIC	artificial saliva (pH 3.4)	―	<0.001	<0.001
	artificial saliva (pH 6.7)	<0.001	―	<0.001
	distilled water (pH 7)	<0.001	<0.001	―

## DISCUSSION

The present study evaluated the solubility of three commonly used luting cements: SeT, Panavia SA Cement, and RMGIC, under varying pH conditions to simulate the environment of the oral cavity. The findings revealed that all three cements exhibited significantly increased solubility under highly acidic conditions (pH 3.4), with Panavia SA showing the least solubility (0.022 ± 0.010), while SeT and RMGIC demonstrated markedly higher values (0.08 ± 0.021 and 0.08 ± 0.005), respectively. However, at neutral and near-neutral pH levels (6.7 and 7.0), no significant solubility differences were observed among the materials. These findings are consistent with the results reported by Eriwati YK et al.^14^, who noted that Conventional GICs tend to be more soluble in artificial saliva compared to RMGICs. In this study, acidic environments exacerbated solubility in all materials, affirming the susceptibility of glass ionomer-based cements to acid dissolution. Similarly, Toledano et al.^8^ documented higher solubility and water sorption values for RMGIC compared to resin cements, attributing this to the presence of hydrophilic components like HEMA and reduced filler content. Another study also supported that the significantly higher solubility of RMGIC is likely due to their hydrophilic properties, primarily attributed to the presence of HEMA in their composition.^15^

The pH plays a critical role in the stability and performance of the luting cements.^16^ A study by Gavranović-Glamoč A et al.^17^ found that RMGIC (GC Fuji Plus) showed significantly higher solubility than the two resin cements (Multilink Automix and Variolink II) across all tested media, reinforcing the vulnerability of RMGIC to acidic environments. Multilink Automix exhibited greater solubility than Variolink II in artificial saliva at pH 3.0 (p < 0.016), while GC Fuji Plus displayed significantly elevated solubility in the same acidic medium (p < 0.009). These findings were corroborated with the present study and revealed that the solubility of all three luting cements significantly increased in response to decreasing pH. The SeT showed the highest sensitivity to pH variations, with statistically significant differences across all three pH levels, indicating that its structural integrity may be compromised in acidic oral conditions.

Panavia SA also demonstrated pH-dependent solubility, albeit to a lesser extent, suggesting better resistance to acid exposure. The low solubility of Panavia SA may be attributed to chemical stability of MDP monomer, enhanced polymer cross-linking. This also suggests that MDP-containing resin cements may be preferable for restorations in patients with acidic oral environments, such as those with xerostomia or high dietary acid intake. Similarly, RMGIC exhibited significantly higher solubility at pH 3.4 than at pH 6.7 or 7.0 (p < 0.001), confirming its susceptibility to degradation in acidic environments. Similarly, these present study’s findings are consistent with those reported by Bharali et al.^18^ who also observed increased solubility of conventional cements, as well as RMGIC and composite cements, when exposed to saliva with a lower pH.

Additionally, the intragroup analysis in the present study demonstrated that each luting cement responded differently across pH levels. The SeT showed significant solubility variation between all pH levels, indicating high pH sensitivity. Panavia SA also exhibited statistically significant differences but to a lesser degree, suggesting better acid resistance. These results support the findings by Yoshida et al.^19^, who concluded that resin cements, particularly those with filler content, perform better in resisting dissolution, exhibit the lowest solubility when compared to glass ionomer cement, and all types of cements show a notable increase in solubility under acidic conditions (pH 4.0). These results are consistent with earlier studies, which also reported that resin cements possess the least solubility among various luting agents.^20^-^23^

Interestingly, Labban N et al.^24^ long-term evaluation of luting agents under water storage over 180 days revealed that RMGICs consistently exhibited the highest water sorption and solubility, with values as high as 4.83% water solubility, while conventional resin cements like Panavia F and Rely X ARC showed the lowest water solubility (0.46-0.67%). In contrast, the present study showed that Panavia SA and similar resin-based cements were more resistant to solubility and degradation, particularly in acidic and moisture-rich environments, making them preferable in high-risk clinical cases. Conversely, RMGICs, despite their fluoride-releasing advantages, remain more vulnerable to acid attack, which may compromise their long-term performance. Moreover, the pH sensitivity of SeT, as demonstrated by significant differences across all pH levels, suggests that formulation variations among resin cements can result in differing susceptibilities. These findings may help clinicians make better decisions when selecting luting materials for patients with low salivary flow or a high risk of caries.

### Limitations

Although the study utilized standardized specimens and pH-controlled artificial saliva, it still had a few limitations. This study was conducted under in vitro conditions, which may not fully replicate the complex oral environment. The use of artificial saliva and static immersion may not account for dynamic factors such as salivary flow, temperature changes, and mechanical stresses. Future research should include in vivo studies to better simulate clinical conditions and evaluate long-term performance. Moreover, in this study, only solubility was evaluated; studies related to properties such as water sorption and bond durability under cyclic loading are also desirable.

## CONCLUSION

The solubility of luting cements is significantly influenced by pH conditions. Among the tested materials, MDP-based resin cement demonstrated superior resistance to acid degradation and may be preferred in patients with acidic oral environments. These findings provide guidance in selecting luting agents for patients with low oral pH or high caries risk.
